# Update of thermotolerant genes essential for survival at a critical high temperature in *Escherichia coli*

**DOI:** 10.1371/journal.pone.0189487

**Published:** 2018-02-27

**Authors:** Masayuki Murata, Ayana Ishii, Hiroko Fujimoto, Kaori Nishimura, Tomoyuki Kosaka, Hirotada Mori, Mamoru Yamada

**Affiliations:** 1 Life Science, Graduate School of Science and Technology for Innovation, Yamaguchi University, Ube, Japan; 2 Department of Biological Chemistry, Faculty of Agriculture, Yamaguchi University, Yamaguchi, Japan; 3 Research Center for Thermotolerant Microbial Resources, Yamaguchi University, Yamaguchi, Japan; 4 Graduate School of Information Science, Nara Institute of Science and Technology, Takayama, Ikoma, Nara, Japan; Universite Paris-Sud, FRANCE

## Abstract

Previous screening of a single-gene knockout library consisting of 3,908 disrupted-mutant strains allowed us to identify 51 thermotolerant genes that are essential for survival at a critical high temperature (CHT) in *Escherichia coli* [Murata M, Fujimoto H, Nishimura K, Charoensuk K, Nagamitsu H, Raina S, Kosaka T, Oshima T, Ogasawara N, Yamada M (2011) PLoS ONE 6: e20063]. In this study, we identified another 21 thermotolerant genes. *E*. *coli* thus has 72 thermotolerant genes in total. The genes are classified into 8 groups: genes for energy metabolism, outer membrane organization, DNA double-strand break repair, tRNA modification, protein quality control, translation control, cell division and transporters. This classification and physiological analysis indicate the existence of fundamental strategies for survival at a CHT, which seems to exclude most of the heat shock responses.

## Introduction

Like general essential genes that are imperative for growth, there are genes, called thermotolerant genes, that are indispensable for survival at a critical high temperature (CHT), a level close to that causing cell death [1, 2]. To understand the molecular mechanisms supporting survival at a CHT in *Escherichia coli*, genome-wide screening with a single-gene knockout library has been performed, and 51 thermotolerant genes essential for growth at 47°C have been identified [1]. Genes for which expression is affected by exposure to a CHT have also been identified [1]. Unexpectedly, the former contents are not consistent with the latter except for *dnaJ* and *dnaK*, indicating that a specific set of non-heat shock genes is required for the organism to survive under such a severe condition. More than half of the mutants of thermotolerant genes are sensitive to H_2_O_2_, suggesting that the mechanism of thermotolerance partially overlaps with that of oxidative stress resistance. Interestingly, gene sets for a lipopolysaccharide biosynthesis system for outer membrane organization and for a sulfur-relay system for tRNA modification are included, and they would have been acquired by horizontal gene transfer.

Here, we update thermotolerant genes in *E*. *coli* and discuss the possible thermotolerant mechanisms for survival at a CHT. Notably, we obtained evidence that ATP synthesis by oxidative phosphorylation is essential at a CHT but not at temperatures below the CHT.

## Materials and methods

### Materials

Oligonucleotide primers for polymerase chain reaction (PCR) were purchased from FASMAC Co, Ltd (Atsugi, Japan). Other chemicals were all of analytical grade.

### Bacterial strains and growth conditions

The strains used in this study were derivatives of *E*. *coli* K-12. BW25113 (*rrnB3*, *ΔlacZ4787*, *hsdR514*, *Δ*(*araBAD)567*, *Δ*(*rhaBAD)568*, *rph-1*) [1] and mutants of BW25113 in the Keio collection as a single-gene knockout library [3] were grown on plates or in liquid of modified Luria-Bertani (LB) medium (1% Bactotryptone, 0.5% yeast extract, and 0.5% NaCl) at various temperatures as described previously [1].

### Effects of glucose and MgCl_2_ and sensitivity to H_2_O_2_

To examine the effects of supplements, glucose (0.5% (w/v)) or MgCl_2_ (20 mM) was added to the LB liquid culture as described in detail previously [1]. The experiments were performed three times, and the results were confirmed to be reproducible.

### RT-PCR analysis

Cultures were grown in LB medium at 37°C until the exponential phase, and then the temperature was up-shifted to 46°C and incubation was continued for 8 min. Total RNA was immediately prepared from the heat-stressed cells by the hot phenol method [1, 4]. RT-PCR analysis was performed using a One-Step RNA PCR kit (AMV) (TAKARA BIO Inc, Otsu, Japan) to examine the expression of immediate downstream genes of disrupted genes as described in detail previously [1, 5]. The primer set used for each gene is shown in S1 Table. After the completion of PCR, the products were analyzed by 1.2% agarose gel electrophoresis and stained with ethidium bromide.

### DNA manipulation

Recombinant plasmids for expression of thermotolerant genes, *atpA*, *gntK*, *lpxL*, *fimG*, *yccM*, *yraN*, *cydD* and *nhaA*, were constructed, generating pUC-atpA, pUC-gntK, pUC-lpxL, pUC-fimG, pUC-yccM, pUC-yraN, pUC-cydD and pUC-nhaA, respectively. The DNA fragment of each gene, including the sequence from 40-bp upstream from the translation initiation codon to the stop codon, was amplified and inserted into pUC19 by In-Fusion cloning (Clontech, USA). Amplification of the DNA fragment was performed by PCR using a specific primer set for each gene (S2 Table) and the genomic DNA of BW25113 as a template [6]. The DNA fragment of pUC19 [7] was also amplified by PCR using two primers, 5'-ACTCTTCCTTTTTCAATATTATTGAAGCA-3' and 5'-CTGTCAGACCAAGTTTACTCATATATAC-3', and pUC19 DNA as a template. The primer set for each thermotolerant gene and that for pUC19 shared 15 overlapping sequences that were required for In-Fusion cloning. Each gene inserted into the vector was designated to be transcribed from the *lac* promoter on the plasmid.

### Complementation test

pUC-atpA, pUC-gntK, pUC-lpxL, pUC-fimG, pUC-yccM, pUC-yraN, pUC-cydD and pUC-nhaA DNAs were introduced into BW25113*atpA*::*kan*, BW25113*gntK*::*kan*, BW25113*lpxL*::*kan*, BW25113*fimG*::*kan*, BW25113*yccM*::*kan*, BW25113*yraN*::*kan*, BW25113*cydD*::*kan* and BW25113*nhaA*::*kan* cells, respectively. The empty plasmid DNA of pUC19 was also introduced into each of these mutant strains and BW25113, which were used as controls. These transformants were grown in LB medium at 37°C or 45°C for appropriate times. The experiments were performed three times, and the results were confirmed to be reproducible.

## Results and discussion

### Identification of new thermotolerant genes

In a previous study, three successive screening steps were performed in a single-gene knockout library [3], and eventually 51 mutant strains that were sensitive to a CHT were found and reported [1]. In the screening process, 26 strains that exhibited relatively slow growth at 30°C on plates at the 1^st^ screening, which was examined at least twice for confirmation, were removed from further screenings by a spotting test and liquid culture. In this study, the 26 mutants were subjected to the 2^nd^ and 3^rd^ screenings of spotting and liquid culturing tests at a CHT as described previously [1] and careful growth experiments in liquid culture at 30°C, 37°C, 39°C, 44°C, 45°C and 46°C. All of the mutants were found to be significantly sensitive to a temperature of 46°C and some were even sensitive to 44°C (S1 Fig and Table 1). However, 5 of those mutants, *atpB*, *atpC*, *atpE*, *atpF* and *atpH*, that encode constituents in the complex of the F_1_F_o_-type ATP synthase showed little growth even at low temperatures, 30°C, 37°C and 39°C. Thus, out of the corresponding 26 genes, the 5 genes for the ATP synthase were excluded and 21 genes have become new thermotolerant genes in *E*. *coli*. In addition, the effects of supplements on cell growth of the sensitive mutants at a CHT were examined under the same conditions as those used previously [1] (S2 Fig and Table 1). Their sensitivity to H_2_O_2_ at 30°C was also tested because about 60% of disrupted mutants of thermotolerant genes are sensitive to H_2_O_2_ [1] (S2 Fig and Table 1). Moreover, according to the gene organization in the local region including the disrupted gene (S3 Fig), a possible polar effect of insertion of the *aph* cassette [3] was examined by RT-PCR with a specific primer set for each gene (S1 Table), but none of the mutants showed an obvious polar effect (S3 and S4 Figs), suggesting that the thermosensitive phenotype in the 21 mutants is due to disruption of the targeted gene. Furthermore, functional complementation was performed on 8 thermotolerant genes selected from five categories that are shown below (S5 Fig). As a result, all of the genes cloned on the plasmid were able to almost completely or partially complement the corresponding gene-knockout mutant. Notably, it was found that introduction of the empty vector, pUC19, and pUC-lpxL caused a negative and a positive effect, respectively, on growth at 37°C in BW25113 *lpxL*::*kan*. Similar but slightly weaker effects were found in the complementation of mutations of *yraN*, *yccM* and *nhaA*. It is likely that these mutations are sensitive to the presence of pUC19 and that each plasmid clone of these genes not only complements the corresponding mutation but also gives resistance to the vector. The mechanism of the growth influence by pUC19, however, is not obvious.

**Table 1:** Thermotolerant genes identified in this study.

Classification	Sub-classification	Gene	Function	30°C^a^	37°C^b^	44°C^c^	45°C^d^	46°C^e^	Glc^f^	Mg^2+^^g^	H_2_O_2_^h^
Energy metabolism (Group A)	Oxidative phosphorylation	*atpA*	F1 sector of membrane-bound ATP synthase, alpha subunit					-			
	Oxidative phosphorylation	*atpD*	F1 sector of membrane-bound ATP synthase, beta subunit					-	+	++	S
	Oxidative phosphorylation	*atpG*	F1 sector of membrane-bound ATP synthase, gamma subunit					-			
	Oxidative phosphorylation	*nuoC*	NADH:ubiquinone oxidoreductase, chain C, D					-			
	Pentose phosphate pathway	*gntK*	Gluconate kinase 2					-		+	S
	Pentose phosphate pathway	*phnN*	Ribose 1,5-bisphosphokinase					-			
	Ubiquinone/menaquinone biosynthesis	*ubiE*	Ubiquinone/menaquinone biosynthesis C-methyltransferase UbiE					-			
	Ubiquinone biosynthesis	*ubiH*	2-octaprenyl-6-methoxyphenol hydroxylase			-	-	-			S
	Ubiquinone biosynthesis	*ubiX*	Flavin prenyltransferase UbiX					-			
	Amino acid metabolism	*dapF*	Diaminopimelate epimerase			-	-	-			
	Amino acid metabolism	*trpB*	Tryptophan synthase, beta chain					-	+		S
	Vitamin B6 metabolism	*pdxH*	Pyridoxin/pyridoxamine 5'-phosphate oxidase				-	-	+		S
Outer membrane biosynthesis (Group B)	Lipopolysaccharide biosynthesis	*lpxL*	Lipid A biosynthesis lauroyltransferase				-	-		++	S
	Secretion system	*fimG*	Minor component of type I fimbriae					-		++	S
DNA repair (Group C)	Putative endonuclease	*yraN*	Predicted Mrr Cat superfamily					-		++	S
tRNA modification (Group D)	tRNA modification	*yccM*	Sulfur relay system, 4Fe-4S membrane protein				-	-		+	
Others	ABC transporter	*cydD*	ATP-binding/membrane protein CydD					-	++	++	S
	Sodium-proton antiporter	*nhaA*	Na^+^:H^+^ antiporter, NhaA family					-	+	++	S
	Hypothetical protein	*yjiY*	Putative transporter, Carbon starvation protein CstA superfamily					-			S
	Hypothetical protein	*ydgH*	Uncharacterized deacetylase					-		++	S
	Hypothetical protein	*yaiS*	DUF1471 family periplasmic protein					-			S

^a to e^“-” means very weak growth at indicated temperatures.

^f^ According to the data in S2 Fig, ratios of growth in the presence of glucose to that in the absence of glucose at 46°C were estimated.

“++” and “+” represent more than 2.0 and 1.5–2.0, respectively.

^g^According to the data in S2 Fig, ratios of growth in the presence of MgCl_2_ to that in the absence of MgCl_2_ at 46°C were estimated.

“++” and “+” represent more than 2.0 and 1.5–2.0, respectively.

^h^According to the data in S2 Fig, ratios of growth in the presence of H_2_O_2_ to that in the absence of H_2_O_2_ at 30°C were estimated.

“S” represents less than 0.5.

Next, the 21 genes corresponding to the 21 thermosensitive mutants were classified on the basis of their functions by using public databases including the KEGG pathway database. Interestingly, most of the 21 thermotolerant genes were found to be classified into categories defined in a previous study: energy metabolism, outer membrane stabilization and tRNA modification (Table 1). It is noteworthy that this study allowed us to find lacking pieces in the following system or pathway. Six genes that are involved in the sulfur-relay system for tRNA modification are included in the previous list of thermotolerant genes [1], and *yccM* as the last one in the sulfur-relay system was newly identified. Similarly, *lpxL*, which encodes KDO2-lipid IV(A) lauroyltransferase, is a new thermotolerant gene in the lipopolysaccharide biosynthesis pathway.

The functions of the newly identified thermotolerant genes are summarized in Table 1. Most of them were categorized into group A, including genes for components or their synthesis in the respiratory chain and in the complex of the F_1_F_o_-type ATP synthase and for enzymes involved in the pentose phosphate pathway, the biosynthesis of lysine or phenylalanine/tyrosine/tryptophan and the metabolism of vitamin B6. The remaining genes were members of groups B, C and D. It is noteworthy that disruption of *fimG* for the outer membrane component in type 1 fimbriae, but not disruption of genes for its other components, causes thermosensitivity, suggesting the importance of outer membrane stability. As noticed previously, the growth of mutants that belong to group B or C was partially improved by the addition of Mg^2+^. Examination of the effect of H_2_O_2_ on growth showed that 14 mutants are sensitive to H_2_O_2_, being 67% of 21 newly identified thermotolerant genes (Table 1). This ratio is consistent with that of 51 previously identified ones, suggesting that the mechanism of thermotolerance partially overlaps with that of oxidative stress resistance in *E*. *coli*, as reported previously [1]. Additionally, DNA chip analysis [1] indicate that none of 21 newly identified thermotolerant genes are not up-regulated when cells are transiently exposed to a CHT, like most of previously identified thermotolerant genes.

Notably, functions of the members of group A suggest that ATP synthesis by substrate-level phosphorylation can support cell survival at temperatures up to 46°C but that ATP synthesis by oxidative phosphorylation is essential at a CHT. On the other hand, mutations of genes for subunits interacting with the membrane in the complex of the ATP synthase were found to be very sensitive even to low temperatures compared to other subunits of the complex. Presumably, mutations for the membrane-interacting subunits cause H^+^ leakage in the membrane, resulting in diminishment of membrane potential, which reduces cell survival of the organism. In addition, the dependence of oxidative phosphorylation at a CHT seems consistent with the essentiality of *aceE*, *aceF*, *lpd*, *lipA* and *ackA* at a CHT [1], which are mapped in the pyruvate metabolism pathway from pyruvate to acetyl CoA [8–11] that functions as energy metabolism for production of ATP. Therefore, these findings allow us to speculate that a metabolic flow at a CHT is toward the TCA cycle and increase in respiratory activity.

### Possible mechanisms for survival at a CHT

In total, 72 thermotolerant genes were found in *E*. *coli* (Table 2). Intriguingly, thermotolerant genes in *E*. *coli* include only a small number of heat shock genes and are mostly genes responsible for functions to stabilize the membrane or to assist fundamental metabolism [1]. These functions are essential for survival in a 2–3°C range close to the CHT in *E*. *coli*. Genes categorized as genes responsible for energy metabolism seem to contribute to the synthesis of ATP, which may be required for repairing damage of macromolecules, protein, DNA or RNA. Many genes responsible for outer membrane stabilization and tRNA modification are involved in modification and stabilization of the structure of LPS and tRNA, respectively. Genes responsible for translation control or cell division may avoid or overcome accidents in translation or the cell division process at a CHT. On the other hand, the involvement of genes in DNA double-strand break repair and chaperone/proteinase suggests accumulation of reactive oxygen species (ROS) at a CHT, being consistent with evidence that a higher temperature results in accumulation of more oxidative stress [12] and the finding that mutants of all members in both groups exhibited sensitivity to H_2_O_2_ [1] and Table 1.

**Table 2:** List of thermotolerant genes in *E*. *coli*.

Classification^a^	Gene	Definition	Function
Energy Metabolism (Group A)	*aceE*	Pyruvate metabolism	Pyruvate dehydrogenase compenent E1 decarboxylase component E1
	*aceF*	Pyruvate metabolism	Pyruvate dehydrogenase, dihydrolipoyltransacetylase compenent E2
	*lpd*	Pyruvate metabolism	Lipoamide dehydrogenase, E3 component, subunit of three comlexes
	*lipA*	Pyruvate mechanism	Liponate synthase
	*ackA*	Pyruvate metabolism	Acetate kinase A and propionate kinase 2
	*rpe*	Pentose phosphate pathway	D-ribulose-5-phosphate 3-epimerase
	*cydB*	Respiratory chain	Cytochrome *d* ubiquinol oxidase subunit II
	*yhcB*	Respiratory chain	Cytochrome *d* ubiquinol oxidase subunit III
	***atpA***^b^	**Oxidative phosphorylation**	**F1 sector of membrane-bound ATP synthase, alpha subunit**
	***atpD***	**Oxidative phosphorylation**	**F1 sector of membrane-bound ATP synthase, beta subunit**
	***atpG***	**Oxidative phosphorylation**	**F1 sector of membrane-bound ATP synthase, gamma subunit**
	***nuoC***	**Oxidative phosphorylation**	**NADH:ubiquinone oxidoreductase, chain C, D**
	***gntK***	**Pentose phosphate pathway**	**Gluconate kinase 2**
	***phnN***	**Pentose phosphate pathway**	**Ribose 1,5-bisphosphokinase**
	***ubiE***	**Ubiquinone/menaquinone biosynthesis**	**Ubiquinone/menaquinone biosynthesis C-methyltransferase UbiE**
	***ubiH***	**Ubiquinone biosynthesis**	**2-octaprenyl-6-methoxyphenol hydroxylase**
	***ubiX***	**Ubiquinone biosynthesis**	**Flavin prenyltransferase UbiX**
	***dapF***	**Amino acid metabolism**	**Diaminopimelate epimerase**
	***trpB***	**Amino acid metabolism**	**Tryptophan synthase, beta subunit**
	***pdxH***	**Vitamin B6 metabolism**	**Pyridoxine/pyridoxamine 5'-phosphate oxidase**
	*ybhH*	Hypothetical protein	Putative isomerase
Outer membrane stabilization (Group B)	*gmhB*	Lipopolysaccharide biosynthesis	D,D-heptose 1,7-bisphosphate phosphatase
	*lpcA* (*gmhA*)	Lipopolysaccharide biosynthesis	D-sedoheptulose 7-phosphate isomerase
	*rfaC* (*waaC*)	Lipopolysaccharide biosynthesis	ADP-heptose:LPS heptosyl transferase I
	*rfaD* (*waaD*)	Lipopolysaccharide biosynthesis	ADP-L-glycero-D-mannoheptose-6-epimerase, NAD(P)-binding
	*rfaE* (*gmhC*)	Lipopolysaccharide biosynthesis	Fused heptose7-phosphate kinase and heptose 1-phosphate adenyltransferase
	*rfaF* (*waaF*)	Lipopolysaccharide biosynthesis	ADP-heptose:LPS heptosyltransferase II
	*rfaG* (*waaG*)	Lipopolysaccharide biosynthesis	Glucosyltransferase I
	***lpxL***	**Lipopolysaccharide biosynthesis**	**Lipid A biosynthesis lauroyltransferase**
	*ydcL*	Peptidoglycan-associated lipoprotein	Predicted lipoprotein
	*yfgL*	Peptidoglycan-associated lipoprotein	Protein assembly complex, lipoprotein component
	*ynbE*	Peptidoglycan-associated lipoprotein	Predicted lipoprotein
	*nlpI*	Peptidoglycan-associated lipoprotein	Conserved protein
	*ycdO*	Peptidoglycan-associated lipoprotein	Conserved protein
	*pal*	Outer membrane integrity	Tol/Pal system, peptidoglycan-associated outer membrane lipoprotein
	*tolQ*	Outer membrane integrity	Tol/Pal system, membrane-spanning protein
	*tolR*	Outer membrane integrity	Tol/Pal system, membrane-spanning protein
	*yciM*	Outer membrane integrity	Conserved hypothetical protein
	***fimG***	**Secretion system**	**Minor component of type 1 fimbriae**
DNA repair (Group C)	*dnaQ*	DNA replication & repair, DSBR	DNA polymerase III subunit, epsilon
	*holC*	DNA replication & repair, DSBR	DNA polymerase III subunit, chi
	*priA*	DNA replication & repair, DSBR	Primosome factor n'
	*ruvA*	DNA replication & repair, DSBR	Component of RuvABC resolvasome, endonuclease
	*ruvC*	DNA replication & repair, DSBR	Conserved protein required for cell growth
	***yraN***	**Hypothetical protein**	**Predicted Mrr Cat superfamily**
tRNA modification (Group D)	*iscS*	tRNA modification	Sulfer relay system, cysteine desulfurase
	*yheL* (*tusB*)	tRNA modification	Sulfer relay system, predicted intracellular sulfur oxidation protein
	*yheM* (*tusC*)	tRNA modification	Sulfer relay system, predicted intracellular sulfur oxidation protein
	*yheN* (*tusD*)	tRNA modification	Sulfer relay system, predicted intracellular sulfur oxidation protein
	*yhhP* (*tusA*)	tRNA modification	Sulfer relay system, conserved protein required for cell growth
	***yccM***	**tRNA modification**	**Sulfur relay system, 4Fe-4S membrane protein**
	*miaA*	tRNA modification	Delta(2)-isopentenylpyrophosphate tRNA-adenosine transferase
	*trmU*	tRNA modification	tRNA (5-methylaminomethyl-2-thiouridylate)-methyltransferase
	*truA*	tRNA modification	Pseudouridine synthase A
Chaperone /protease (Group E)	*dnaJ*	Chaperone system	Chaperone Hsp40, co-chaperone with DnaK
	*dnaK*	Chaperone system	Chaperone Hsp70, co-chaperone with DnaJ
	*degP*	Chaperone system	Chaperone/serine endoprotease
	*rseA*	Chaperone regulator	Anti-sigma factor
Translational control (Group F)	*rpmJ*	Translational control	50S ribosomal subunit L36, related to *secY* expression
	*rpsF*	Translational control	30S ribosomal subunit S6, modified with glutamic acid or phosphate
	*dksA*	Translational control	DNA-binding transcriptional regulator or rRNA transcription
	*smpB*	Translational control	Component of trans-translation process
Cell division (Group G)	*xerC*	Related to cell division	Site-specific tyrosine recombinase for chromosome dimmer resolution
	*dedD*	Related to cell division	Membrane-anchored periplasmic protein involved in separation
	*envC*	Related to cell division	Regulator of cell wall hydrolases responsible for cell separation
Transporter (Group H)	*zntA*	Membrane transport	Zinc/cadmium/mercury/lead-exporting ATPase
	*ybgH*	Membrane transport	Predicted proton-dependent oligopeptide transporter, POT family
	***cydD***	**Membrane transport**	**ATP-binding/permease protein CydD**
	*nhaA*	**Membrane transport**	**Na**^**+**^**/H**^**+**^ **antiporter, NhaA family**
	***yjiY***	**Hypothetical protein**	**Putative transporter, carbon starvation protein CstA superfamily**
Others	***yaiS***	**Hypothetical protein**	**Uncharacterized deacetylase**
	***ydgH***	**Hypothetical protein**	**DUF1471 family periplasmic protein**

^a^A new group of transporter (Group H) is added. Transporters were classified in others in previous paper [1].

^b^Thermotolerant genes identified in this study are shown by bold letters.

**Fig 1:**
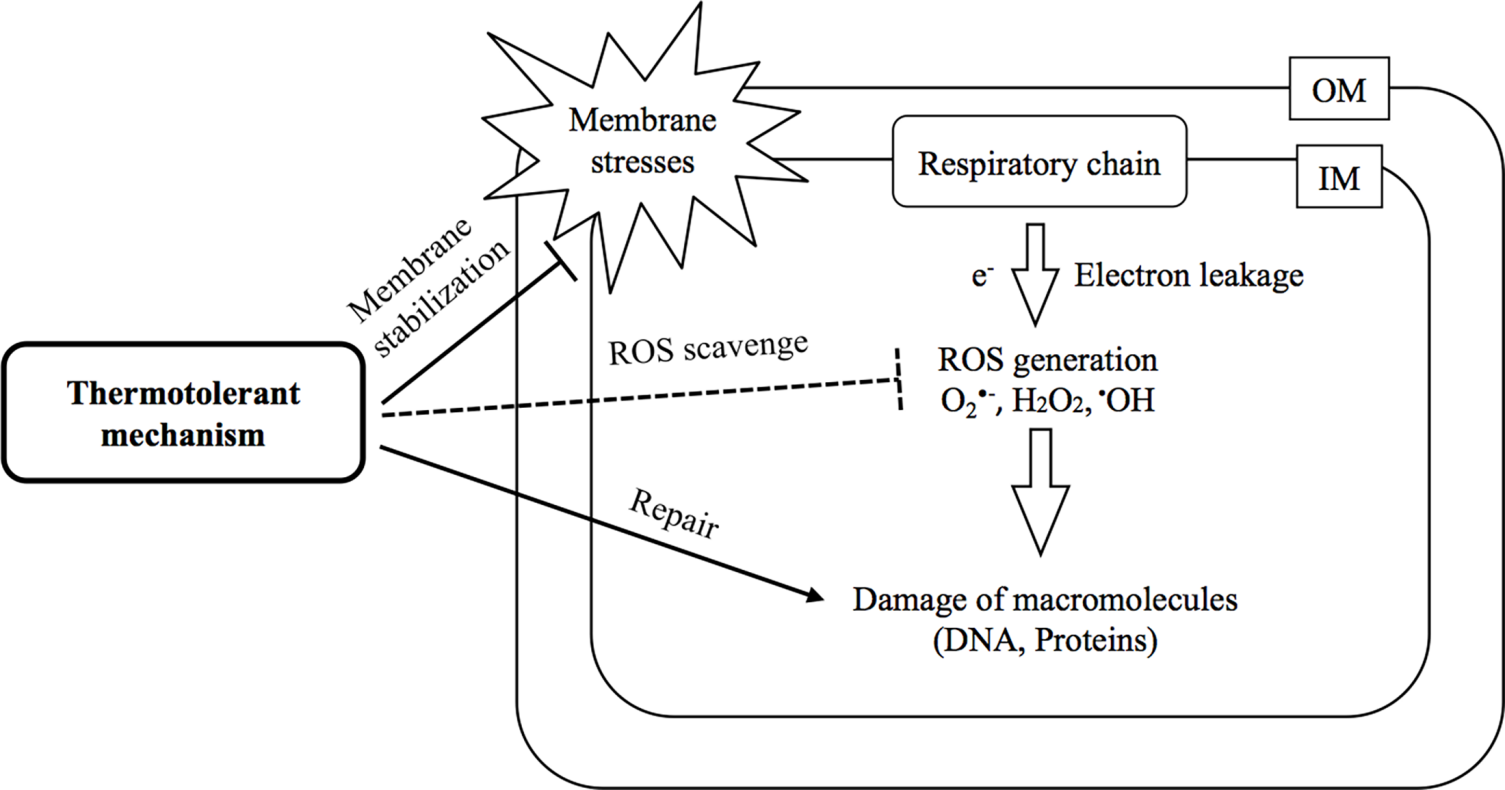
A model of intracellular problems at a CHT and their protection mechanisms. At a CHT, the level of ROS is increased as described in the text, resulting in damage of macromolecules. There are various possible protection mechanisms as thermotolerant mechanisms, such as stabilization of the membrane to protect electron leakage from the respiratory chain, scavenging ROS, and stabilization of tRNA by modification and repair of DNA double-strand breaks or denatured proteins. Abbreviations used are: OM, outer membrane; IM, inner membrane; O_2_^•-^, superoxide radical anion; H_2_O_2_, hydrogen peroxide; ^•^OH, hydroxyl radical.

Taken together, we propose a model of intracellular problems at a CHT and their protection mechanisms as shown in Fig 1. Under conditions at high temperatures close to a CHT, membrane fluidity may dramatically increase, causing leakage of electrons from the respiratory chain to generate ROS that give rise to oxidative damages to macromolecules. DNA double-strand breaks and protein denaturation by oxidative modifications seem to occur in the cytoplasm or periplasm because the corresponding genes become indispensable at a CHT. As protection mechanisms against these problems as thermotolerant mechanisms, LPS and membrane proteins may strengthen the membrane structure to prevent membrane fluidity. However, ROS scavenging genes were not found as thermotolerant genes. This may be due to the existence of homologues that can perform the same function in *E*. *coli*. Notably, overexpression of *sodA* for superoxide dismutase or *katE* for catalase significantly increased the number of viable and culturable cells at a CHT [12]. Similarly, overexpression of catalase and superoxide dismutase genes increased the degree of thermotolerance in *Saccharomyces cerevisiae* [13]. Notably, the generation of ROS might be enhanced by an increase in respiratory activity, as speculated above, for oxidative phosphorylation at a CHT. In addition to membrane stabilization and scavenging of ROS, there are protection mechanisms of stabilization of tRNA by modification and repair of DNA double-strand breaks or denatured proteins in *E*. *coli*. Moreover, there are other trouble-shooting mechanisms in translation or cell division at a CHT.

Since the screening in this study was performed with a single-gene knockout library covering all non-essential genes in *E*. *coli*, knowledge of physiological functions of the 72 thermotolerant genes will be very beneficial for the genetic conversion of non-thermotolerant to thermotolerant bacteria.

## Supporting information

S1 Fig Growth of thermosensitive mutants in LB liquid culture at different temperatures. Each of the 26 thermosensitive mutant strains (open circles) and the parental strain, BW25113 (closed circles), were grown in 30 ml LB medium at 30°C, 37°C, 39°C, 44°C, 45°C and 46°C. At the times indicated, turbidity at OD_600_ was measured. A, group A; B, group B; C, group C; D, group D; E, group E.
(PDF)

S2 Fig Effects of addition of glucose and MgCl_2_ and sensitivity to H_2_O_2_. Thermosensitive mutant strains are shown by gene names. Growth conditions are described in Materials and Methods. Black and white columns represent turbidity under the conditions with and without supplements (0.5% glucose (A) or 20 mM MgCl_2_ (B)) or 0.5 mM H_2_O_2_ (C).
(PDF)

S3 Fig Gene organizations around genes having either an essential gene or a thermotolerant gene as a just downstream gene. Gene organizations around 19 thermotolerant genes that may have either an essential gene or a thermotolerant gene as a just downstream gene are depicted. Black boxes represent 19 identified thermotolerant genes. Grey boxes represent possible essential or thermotolerant genes. The direction of boxes shows the direction of transcription.
(PDF)

S4 Fig Testing of possible polar effects by *aph* insertion. Total RNA was prepared from cells cultured at 37°C (a) and 46°C (b) and subjected to RT-PCR as described in Materials and Methods. RT-PCR was performed with primers specific for a just downstream gene of each thermotolerant gene to amplify about 500-bp DNA fragments. After RT reaction, PCR was performed for 15, 20, 25 and 30 cycles and each PCR product was electrophoresed on 1.2% agarose gel, followed by staining with ethidium bromide. Arrowheads indicate amplified products by RT-PCR.
(PDF)

S5 Fig Complementation experiments with plasmid clones of representative thermotolerant genes. Transformants with plasmid clones (open circles), BW25113*atpA*::*kan* (pUC-atpA), BW25113*gntK*::*kan* (pUC-gntK), BW25113*lpxL*::*kan* (pUC-lpxL), BW25113*fimG*::*kan* (pUC-fimG), BW25113*yccM*::*kan* (pUC-yccM), BW25113*yraN*::*kan* (pUC-yraN), BW25113*cydD*::*kan* (pUC-cydD) and BW25113*nhaA*::*kan* (pUCNHAA), and transformants with an empty vector (closed circles), BW25113*atpA*::*kan* (pUC19), BW25113*gntK*::*kan* (pUC19), BW25113*lpxL*::*kan* (pUC19), BW25113*fimG*::*kan* (pUC19), BW25113*yccM*::*kan* (pUC19), BW25113*yraN*::*kan* (pUC19), BW25113*cydD*::*kan* (pUC19) and BW25113*nhaA*::*kan* (pUC19), and BW25113 (pUC19) (open triangles) were grown in 30 ml LB medium at 37°C (at left side) and 45°C (at right side), except that cells in the BW25113*lpxL*::*kan* background and its control cells were examined at 37°C (at left side) and 43°C (at right side) because of the negative effect of pUC19 (see related description in the text). At the times indicated, turbidity at OD_600_ was measured.
(PDF)

S1 Table RT-PCR primers used in this study. (DOC)

S2 Table Primers for gene cloning for complementation experiments. (DOC)
