# Supplementary figures and images for: Update of thermotolerant genes essential for survival at a critical high temperature in Escherichia coli
Source: PLoS One. 2018 Feb 27;13(2):e0189487. doi: 10.1371/journal.pone.0189487 (PMC5828445; doi:10.1371/journal.pone.0189487)

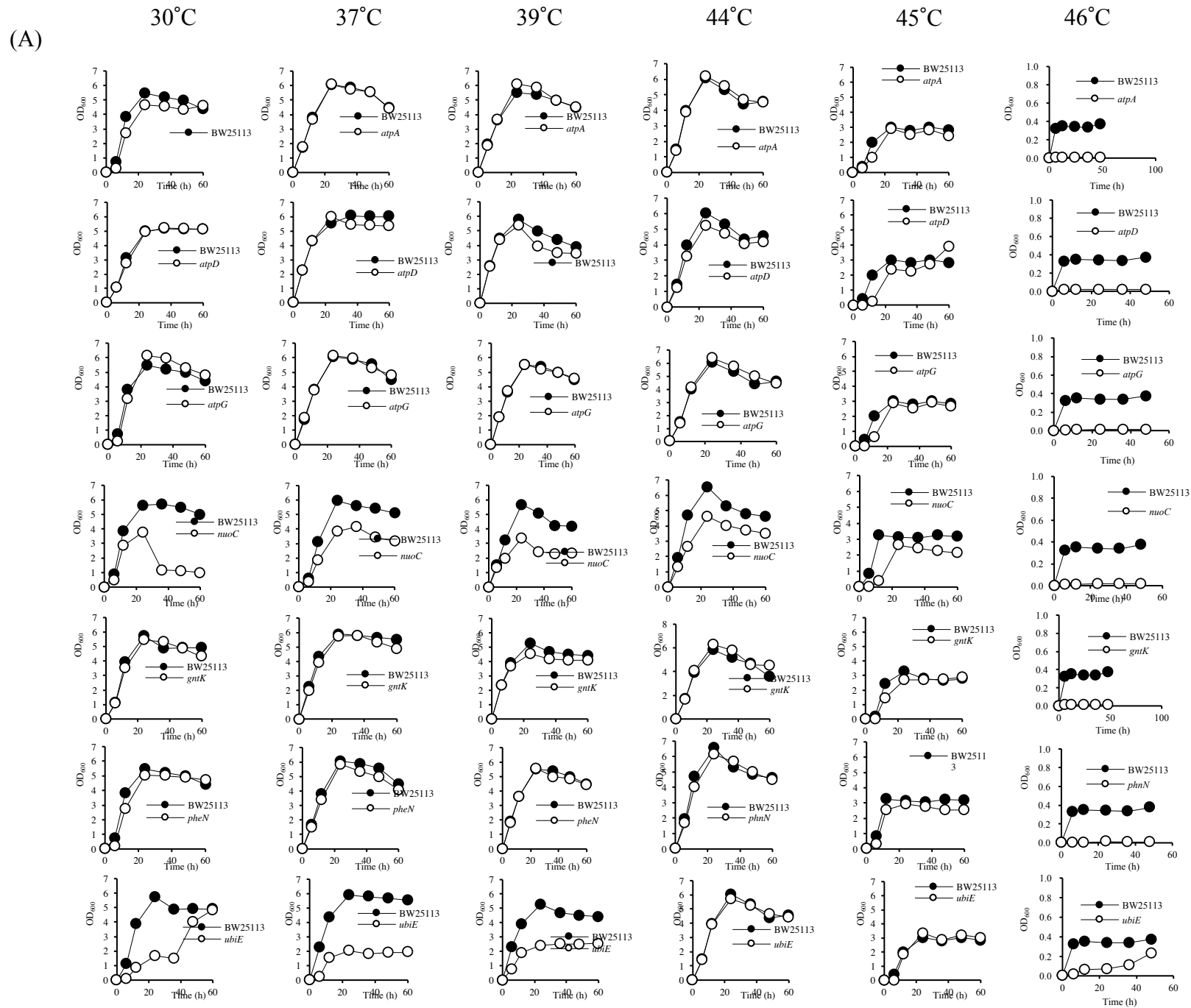



46°C

(D)

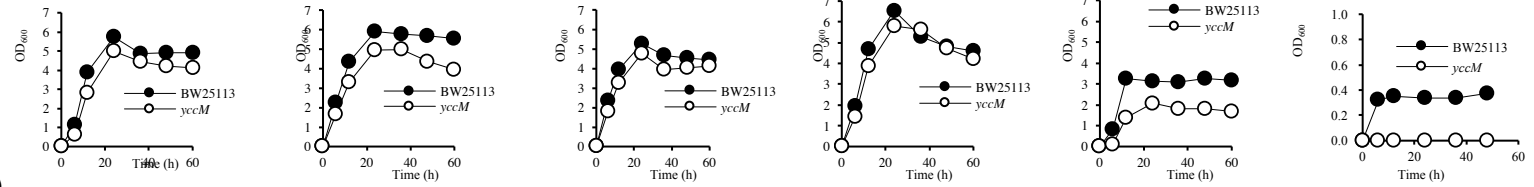

(E)

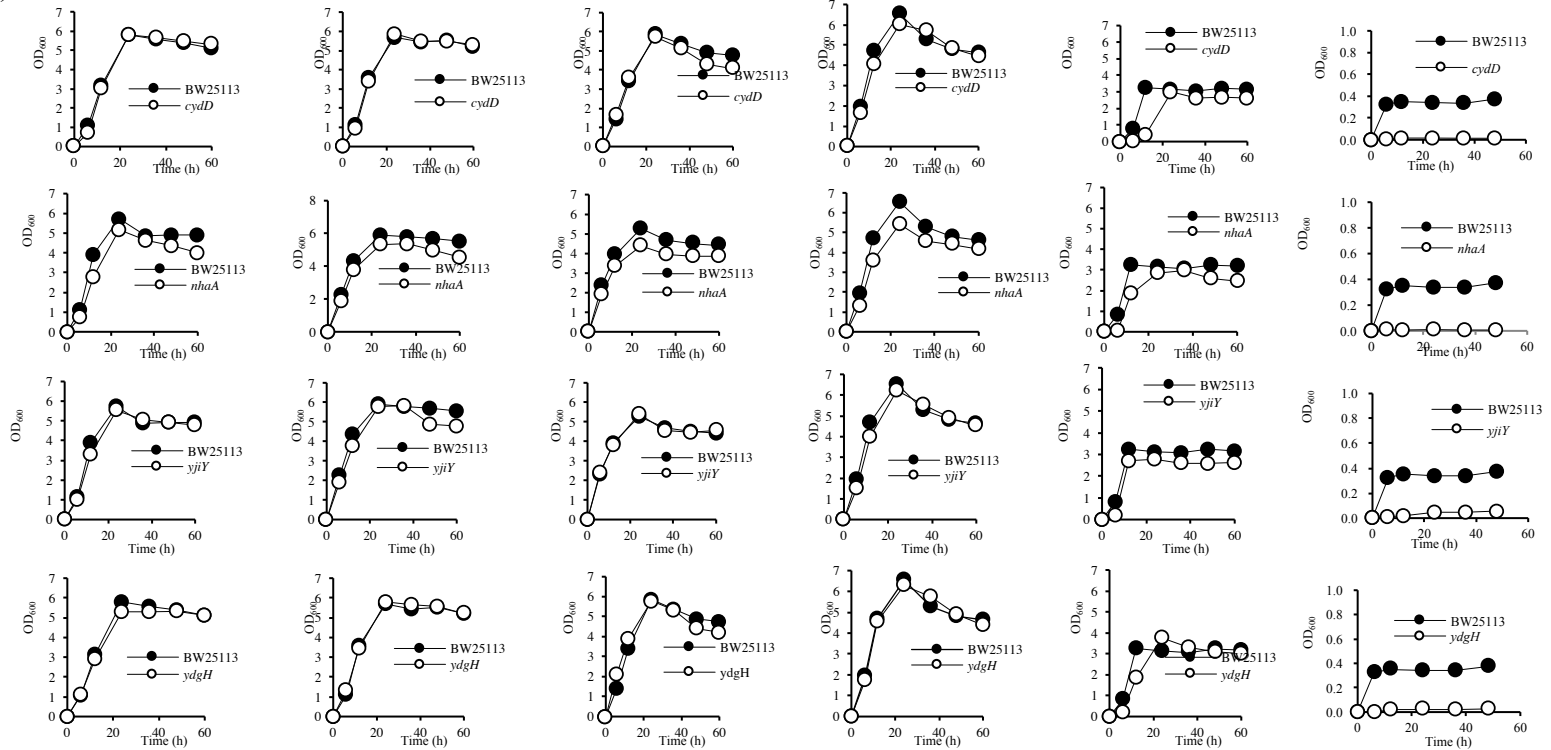

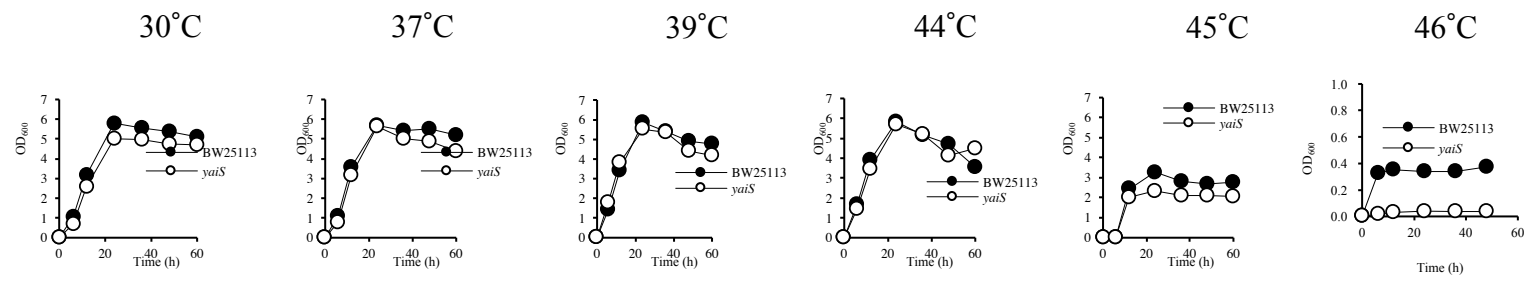

S1 Fig. Murata *et al*

Supplement: S1 Fig — Each of the 26 thermosensitive mutant strains (open circles) and the parental strain, BW25113 (closed circles), were grown in 30 ml LB medium at 30°C, 37°C, 39°C, 44°C, 45°C and 46°C. At the times indicated, turbidity at OD600 was measured. A, group A; B, group B; C, group C; D, group D; E, group E. (PDF) [file pone.0189487.s001.pdf]

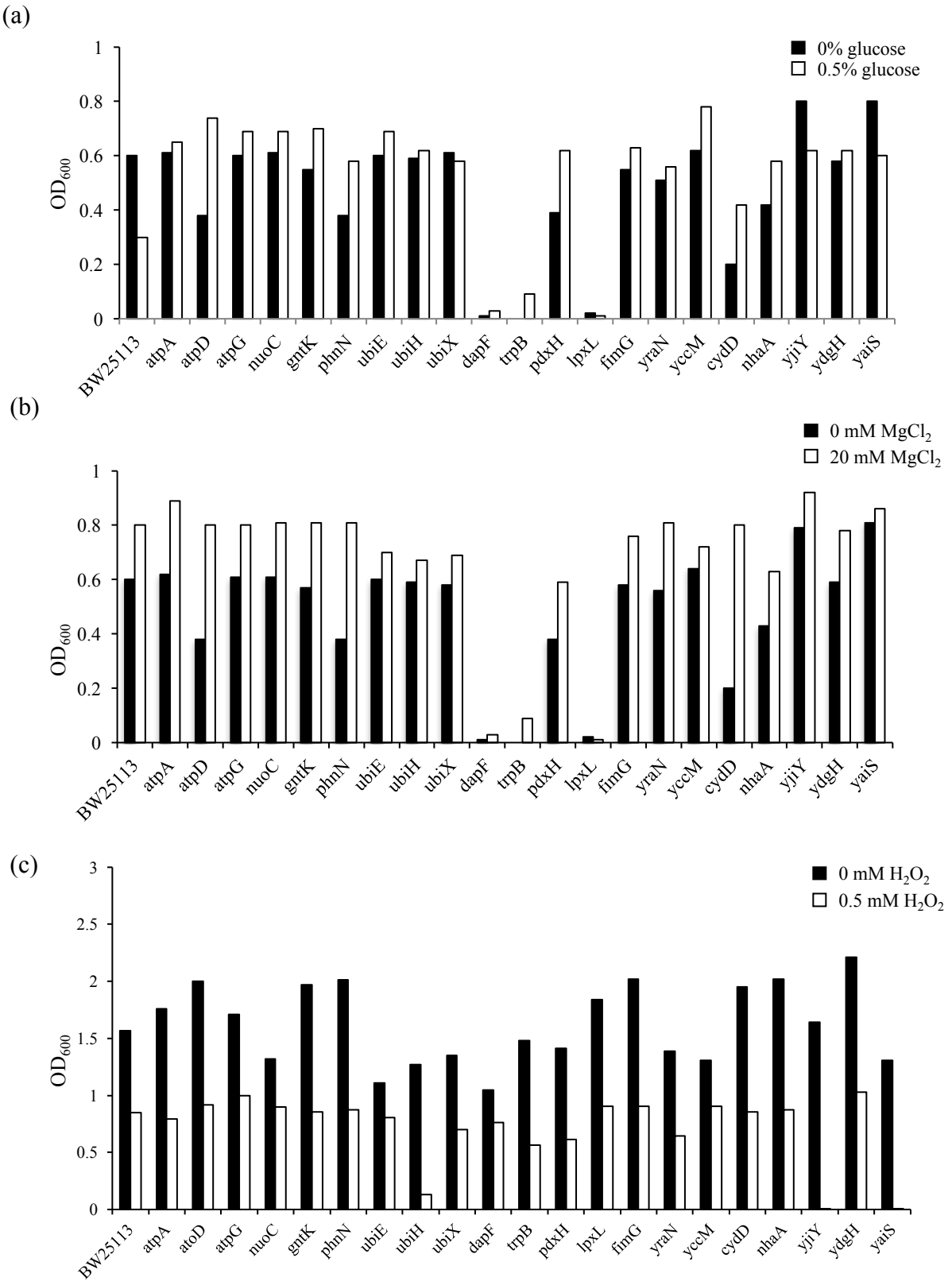

S2 Fig. Murata *et al*

Supplement: S2 Fig — Thermosensitive mutant strains are shown by gene names. Growth conditions are described in Materials and Methods. Black and white columns represent turbidity under the conditions with and without supplements (0.5% glucose (A) or 20 mM MgCl2 (B)) or 0.5 mM H2O2 (C). (PDF) [file pone.0189487.s002.pdf]

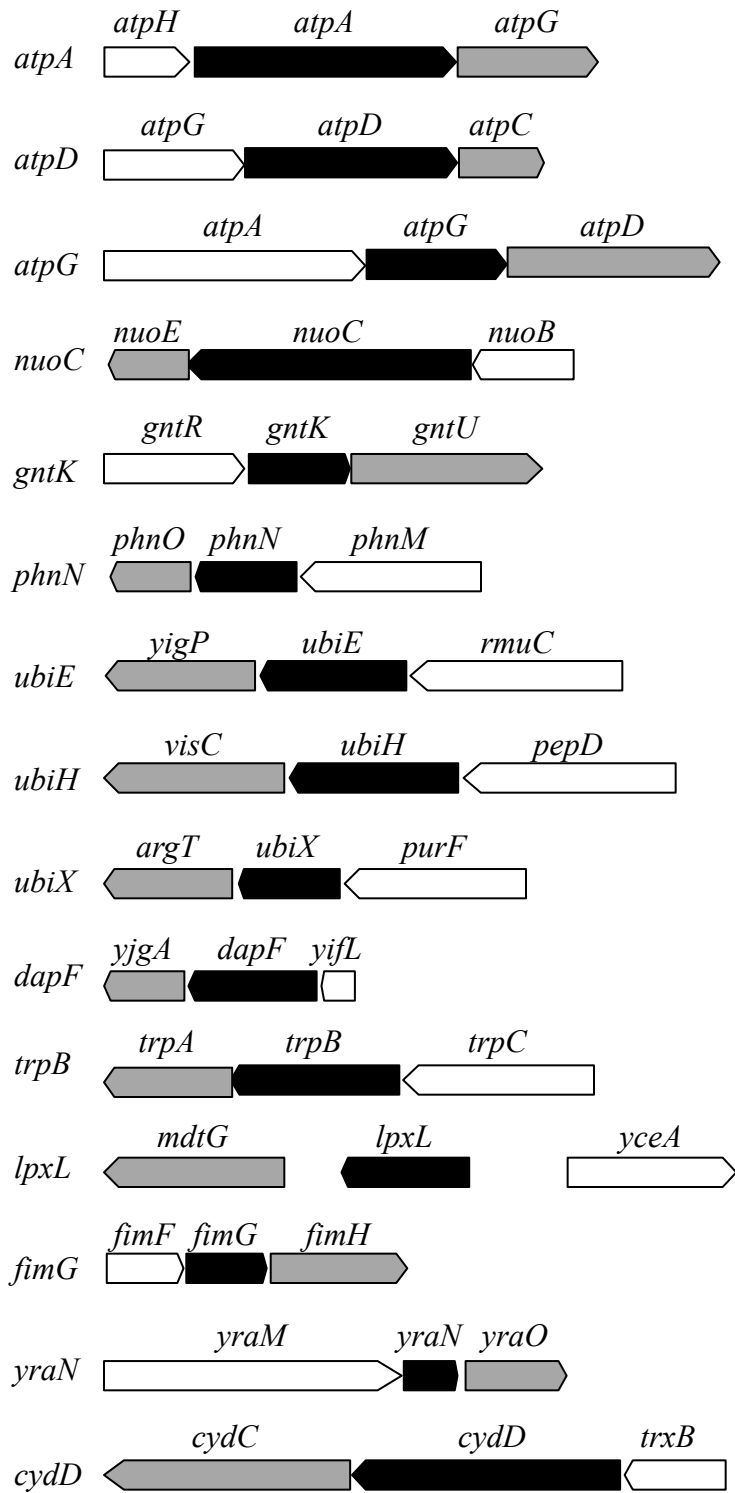

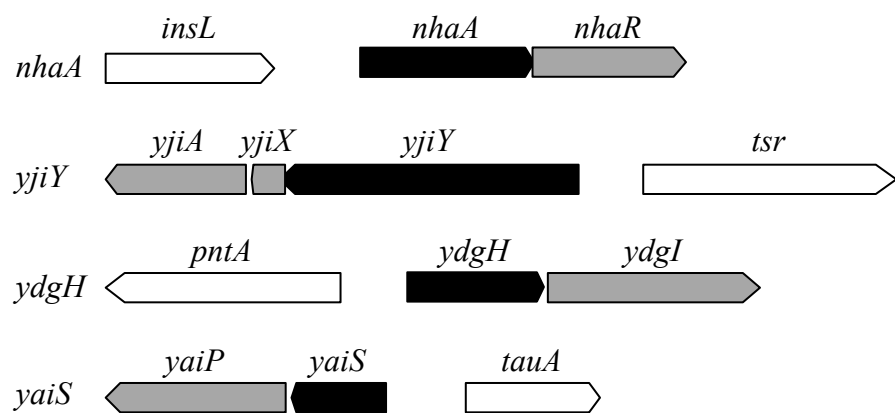

Supplement: S3 Fig — Gene organizations around 19 thermotolerant genes that may have either an essential gene or a thermotolerant gene as a just downstream gene are depicted. Black boxes represent 19 identified thermotolerant genes. Grey boxes represent possible essential or thermotolerant genes. The direction of boxes shows the direction of transcription. (PDF) [file pone.0189487.s003.pdf]

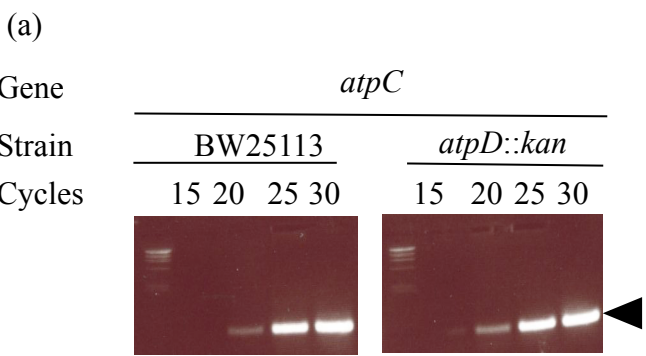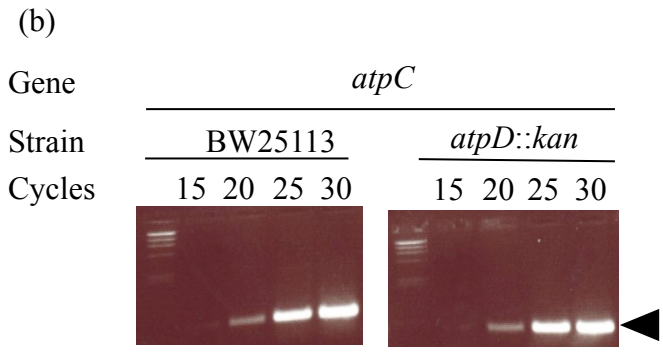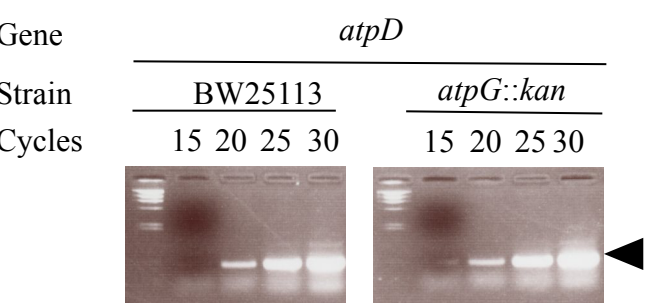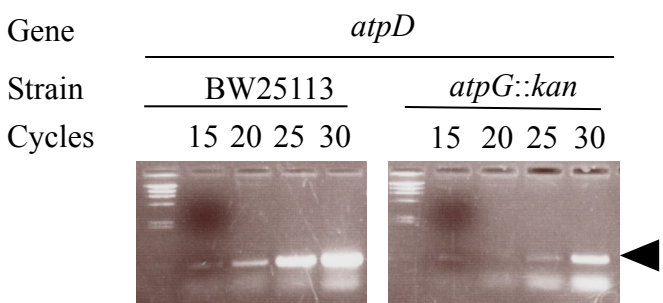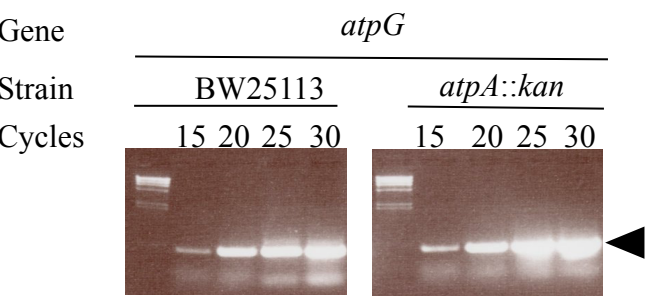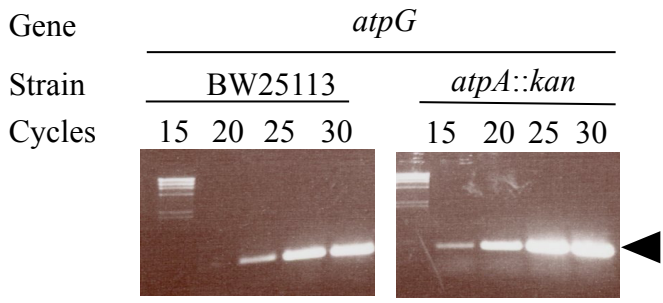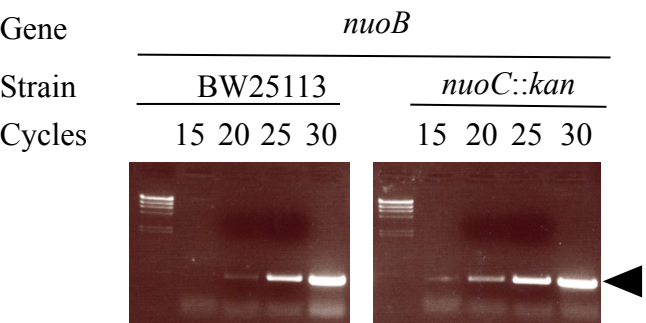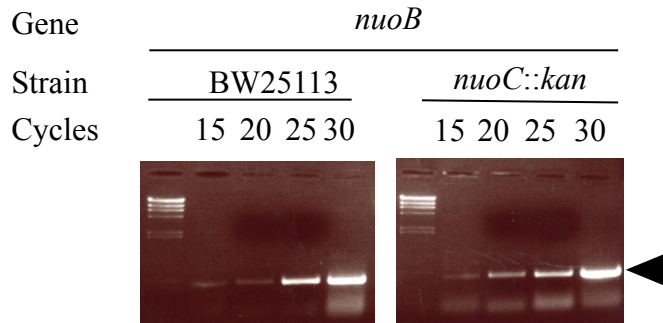

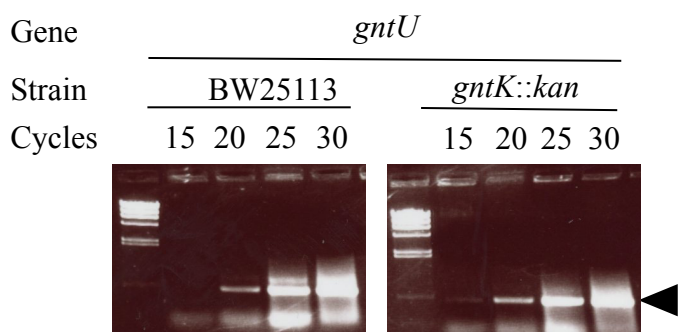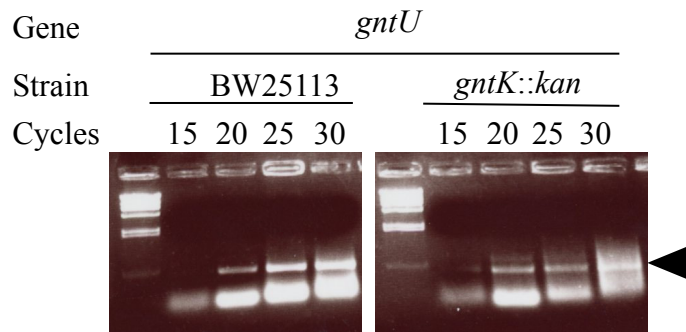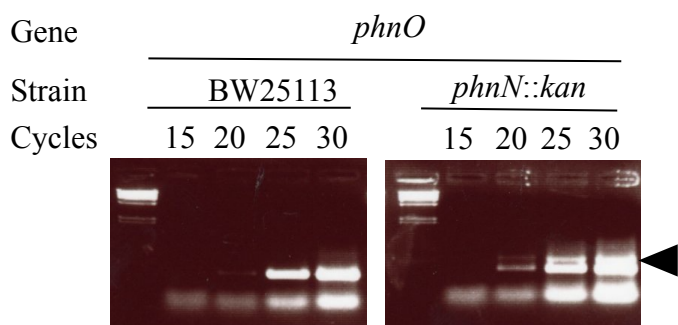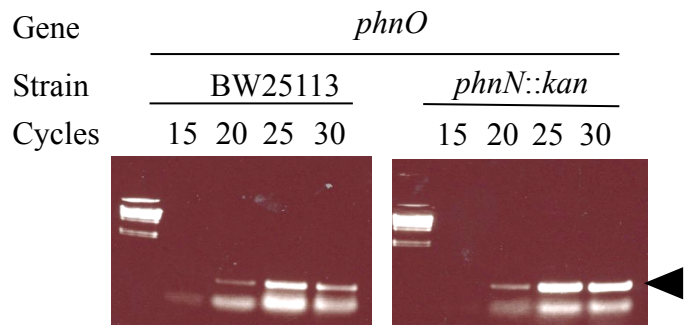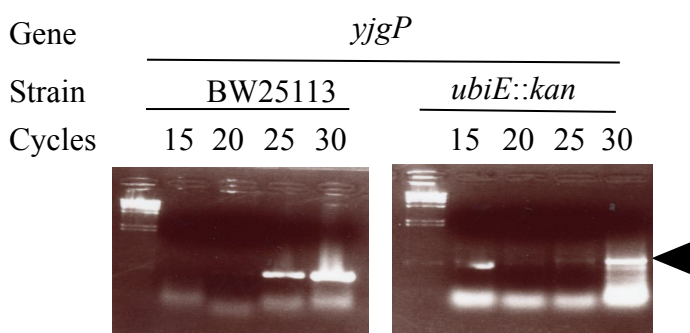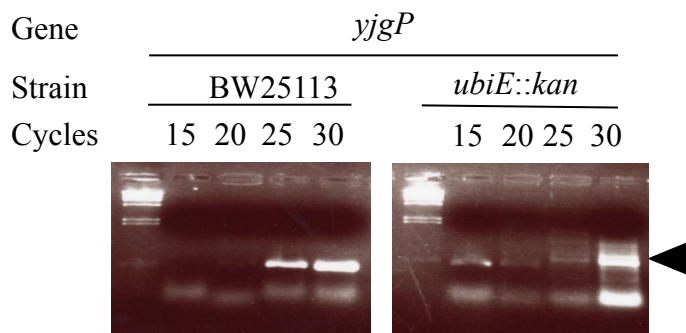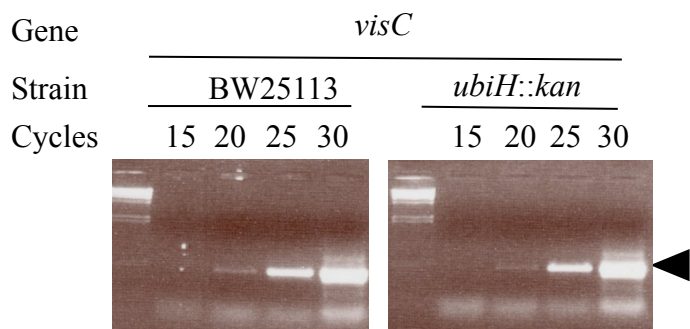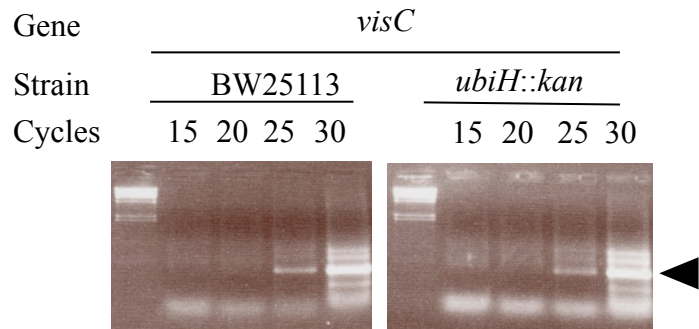

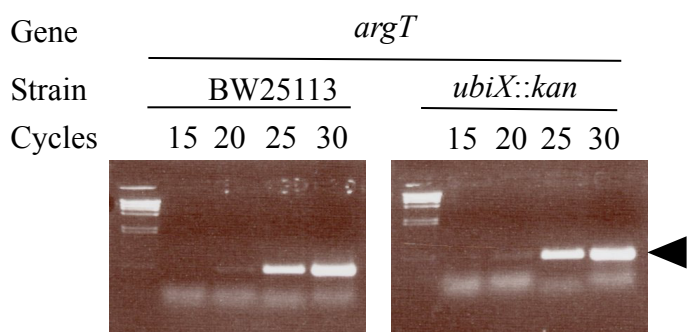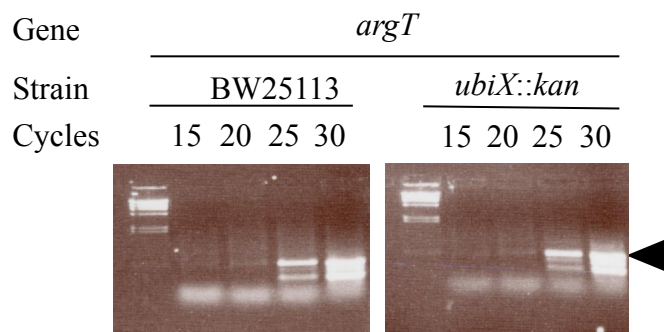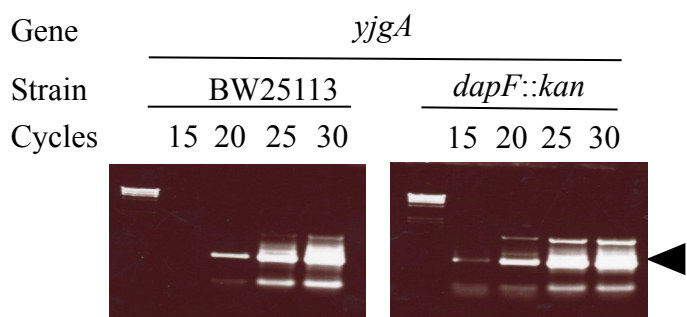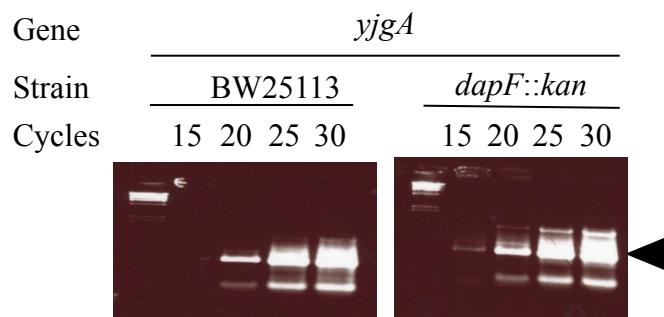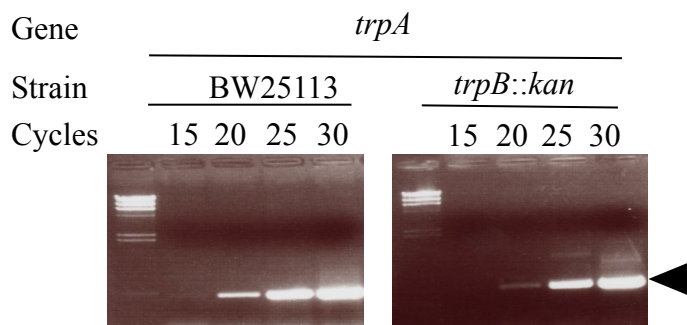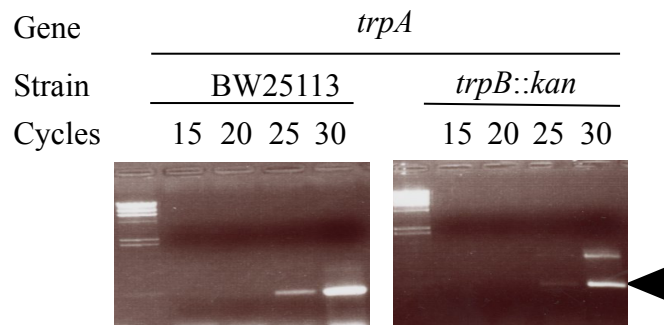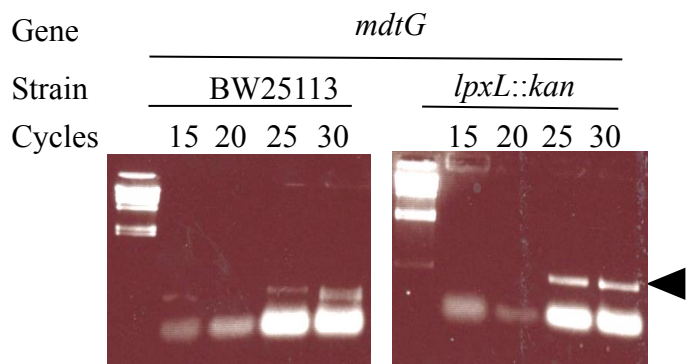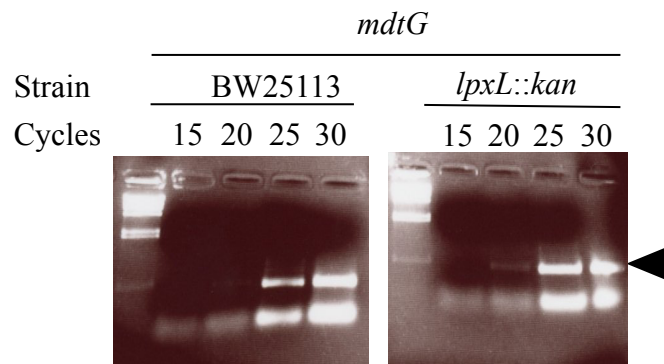

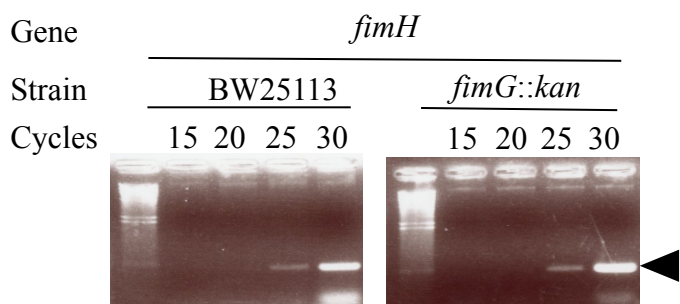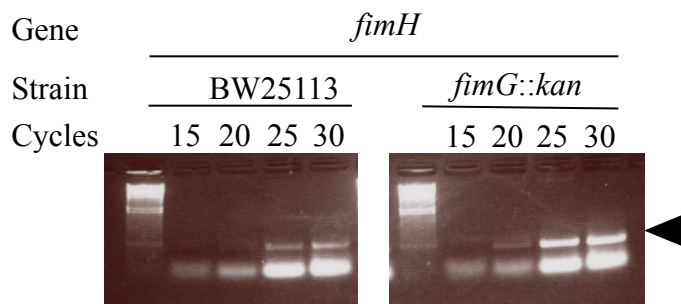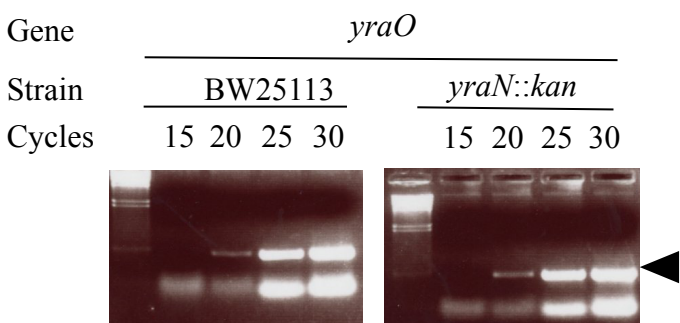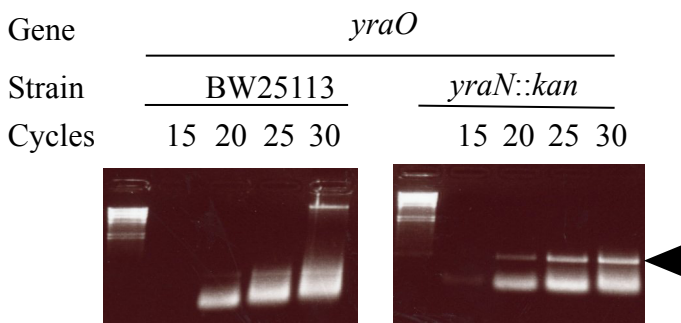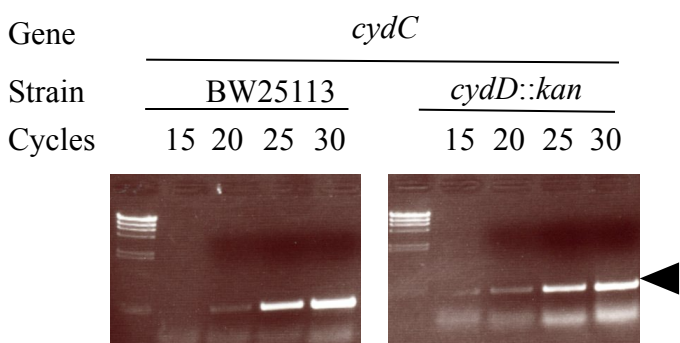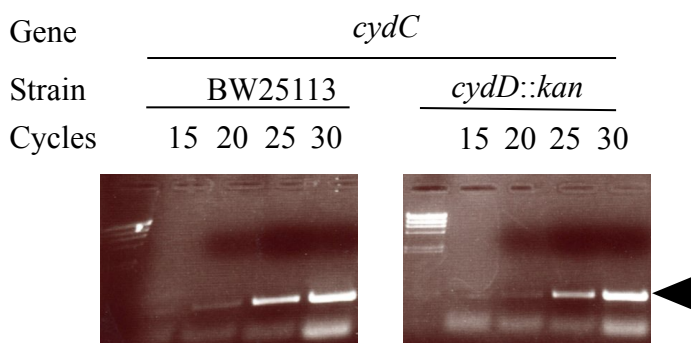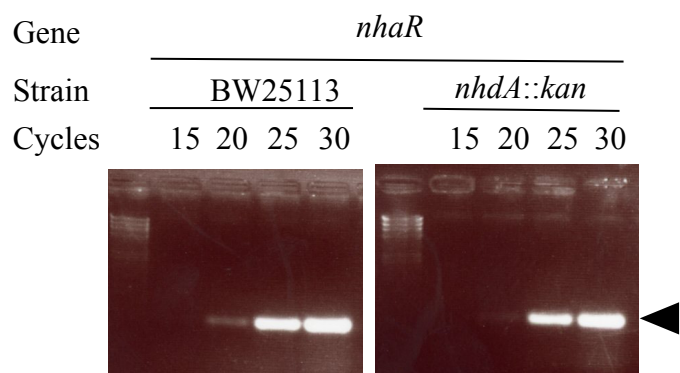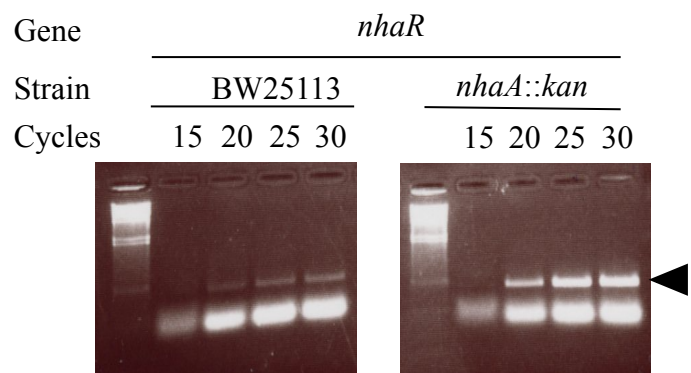

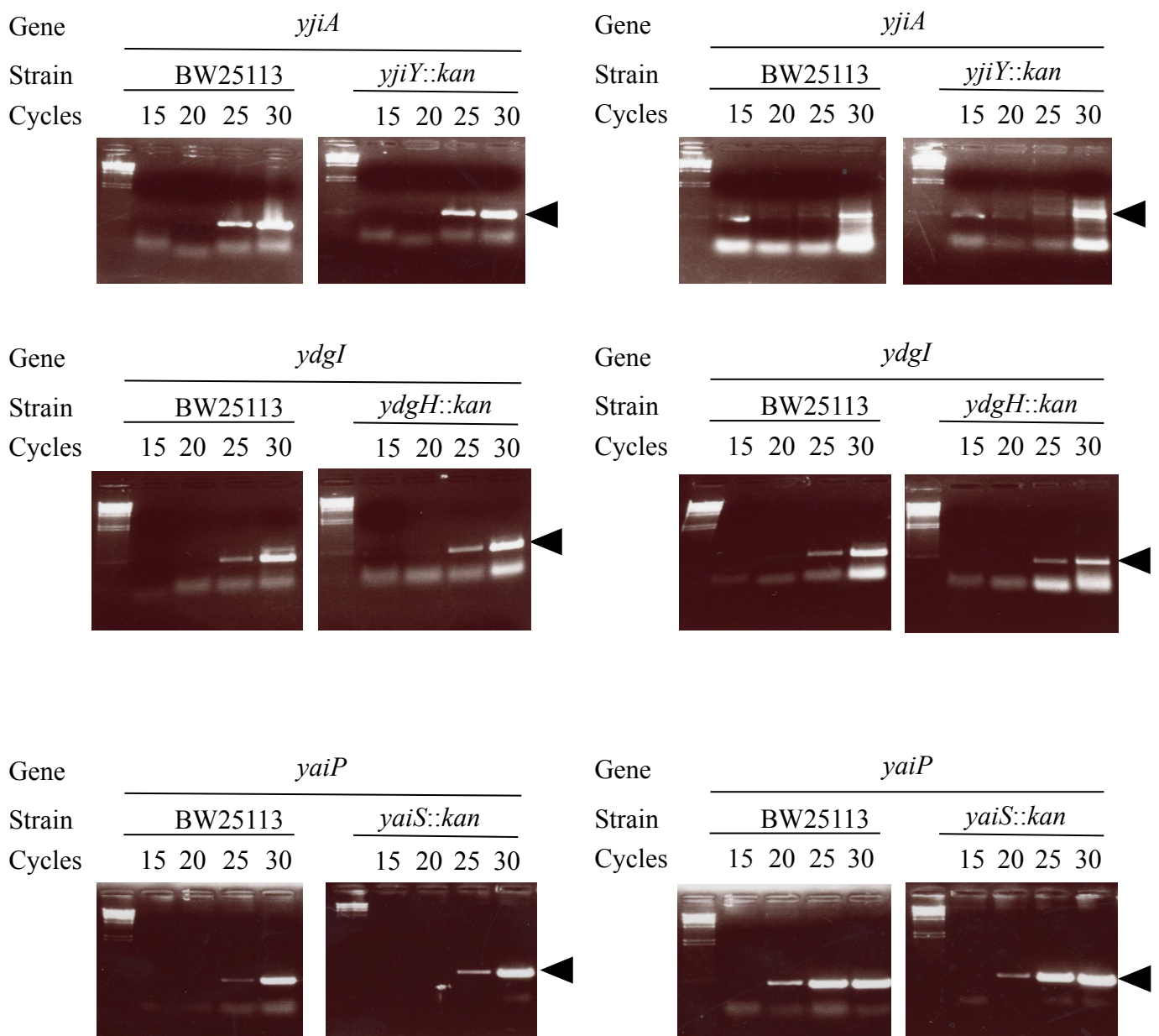

S4 Fig. Murata *et al*

Supplement: S4 Fig — Total RNA was prepared from cells cultured at 37°C (a) and 46°C (b) and subjected to RT-PCR as described in Materials and Methods. RT-PCR was performed with primers specific for a just downstream gene of each thermotolerant gene to amplify about 500-bp DNA fragments. After RT reaction, PCR was performed for 15, 20, 25 and 30 cycles and each PCR product was electrophoresed on 1.2% agarose gel, followed by staining with ethidium bromide. Arrowheads indicate amplified products by RT-PCR. (PDF) [file pone.0189487.s004.pdf]
