# Supplementary material for: Update of thermotolerant genes essential for survival at a critical high temperature in Escherichia coli
Source: PLoS One. 2018 Feb 27;13(2):e0189487. doi: 10.1371/journal.pone.0189487 (PMC5828445; doi:10.1371/journal.pone.0189487)
Supplement: S5 Fig — Transformants with plasmid clones (open circles), BW25113atpA::kan (pUC-atpA), BW25113gntK::kan (pUC-gntK), BW25113lpxL::kan (pUC-lpxL), BW25113fimG::kan (pUC-fimG), BW25113yccM::kan (pUC-yccM), BW25113yraN::kan (pUC-yraN), BW25113cydD::kan (pUC-cydD) and BW25113nhaA::kan (pUCNHAA), and transformants with an empty vector (closed circles), BW25113atpA::kan (pUC19), BW25113gntK::kan (pUC19), BW25113lpxL::kan (pUC19), BW25113fimG::kan (pUC19), BW25113yccM::kan (pUC19), BW25113yraN::kan (pUC19), BW25113cydD::kan (pUC19) and BW25113nhaA::kan (pUC19), and BW25113 (pUC19) (open triangles) were grown in 30 ml LB medium at 37°C (at left side) and 45°C (at right side), except that cells in the BW25113lpxL::kan background and its control cells were examined at 37°C (at left side) and 43°C (at right side) because of the negative effect of pUC19 (see related description in the text). At the times indicated, turbidity at OD600 was measured. (PDF) [file pone.0189487.s005.pdf]

(A)

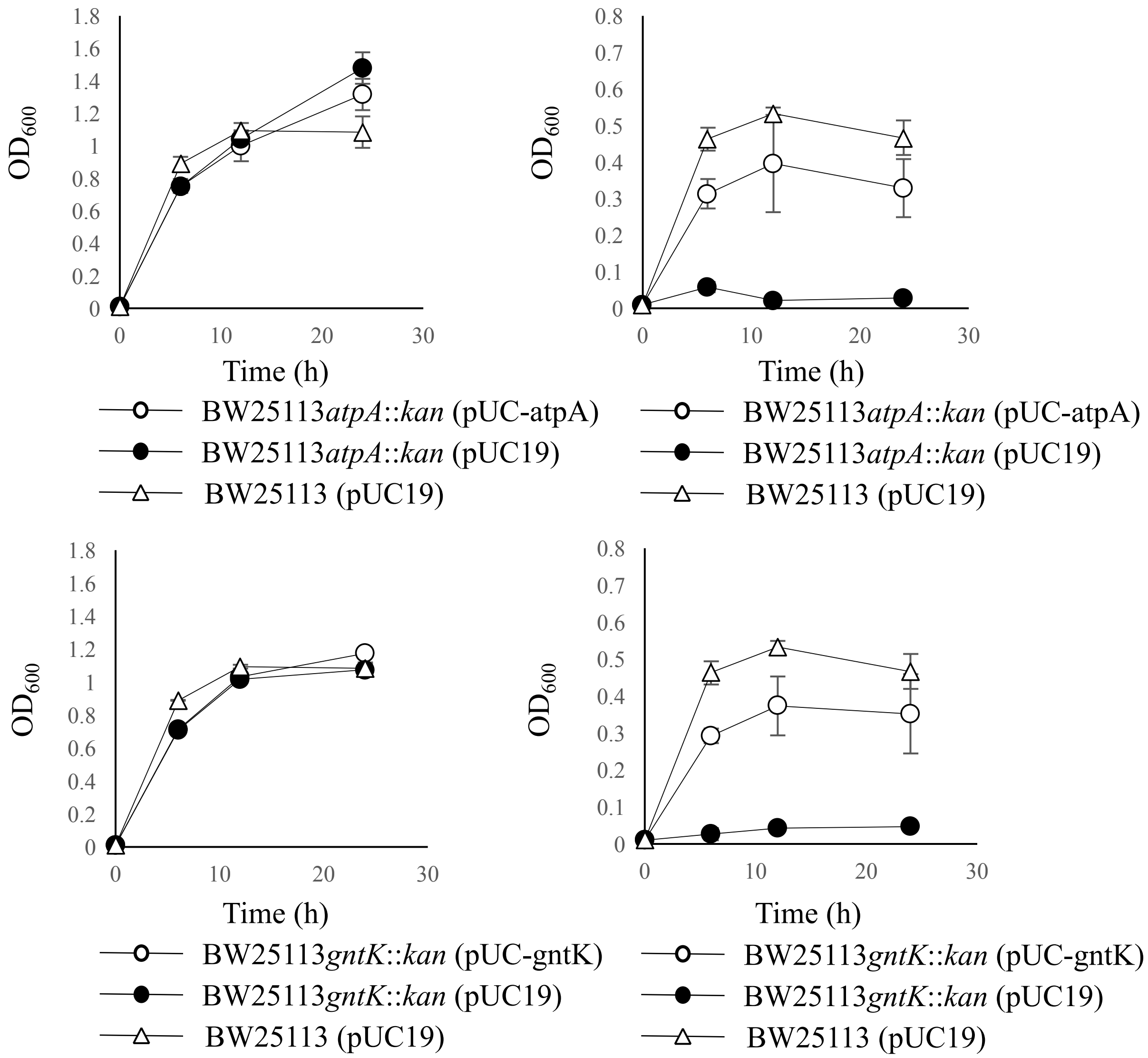

OD600
Time (h)
BW25113atpA::kan (pUC-atpA)
BW25113atpA::kan (pUC19)
BW25113 (pUC19)
OD600
Time (h)
BW25113atpA::kan (pUC-atpA)
BW25113atpA::kan (pUC19)
BW25113 (pUC19)
OD600
Time (h)
BW25113gntK::kan (pUC-gntK)
BW25113gntK::kan (pUC19)
BW25113 (pUC19)
OD600
Time (h)
BW25113gntK::kan (pUC-gntK)
BW25113gntK::kan (pUC19)
BW25113 (pUC19)

(B)

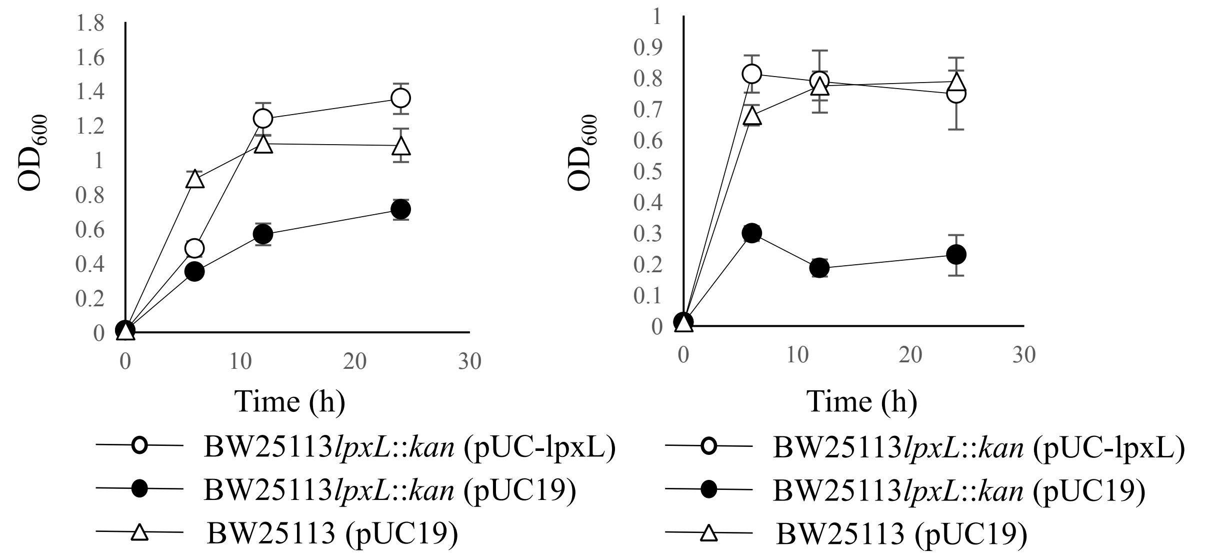

OD600
Time (h)
BW25113lpxL::kan (pUC-lpxL)
BW25113lpxL::kan (pUC19)
BW25113 (pUC19)
OD600
Time (h)
BW25113lpxL::kan (pUC-lpxL)
BW25113lpxL::kan (pUC19)
BW25113 (pUC19)

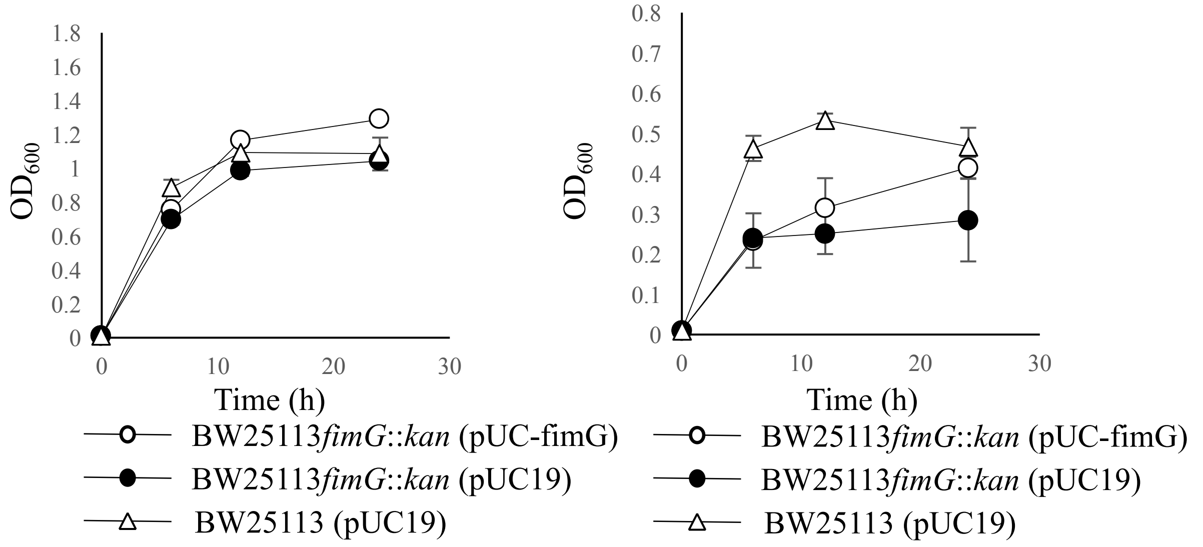
OD600
Time (h)
BW25113fimG::kan (pUC-fimG)
BW25113fimG::kan (pUC19)
BW25113 (pUC19)
OD600
Time (h)
BW25113fimG::kan (pUC-fimG)
BW25113fimG::kan (pUC19)
BW25113 (pUC19)

(C)

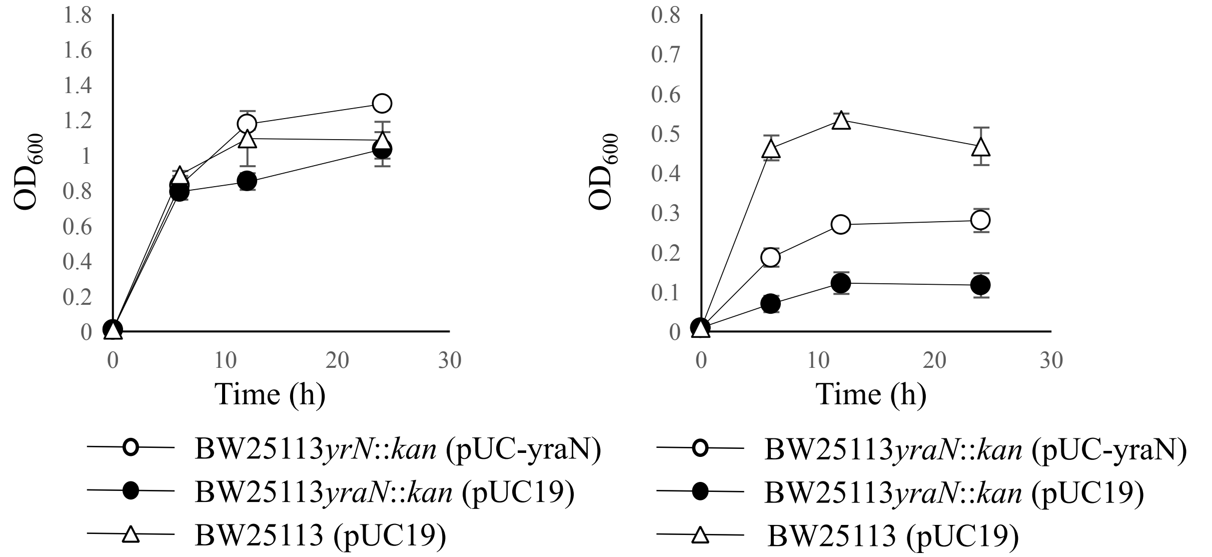
OD600
Time (h)
BW25113yrN::kan (pUC-yraN)
BW25113yraN::kan (pUC19)
BW25113 (pUC19)
OD600
Time (h)
BW25113yraN::kan (pUC-yraN)
BW25113yraN::kan (pUC19)
BW25113 (pUC19)

(D)

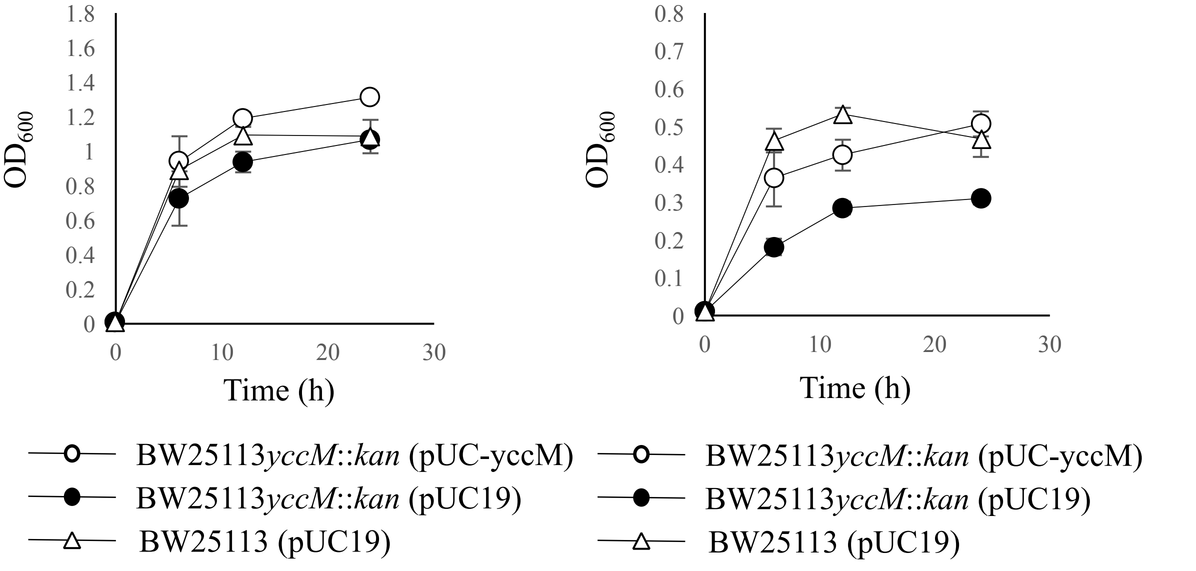
OD600
Time (h)
BW25113yccM::kan (pUC-yccM)
BW25113yccM::kan (pUC19)
BW25113 (pUC19)
OD600
Time (h)
BW25113yccM::kan (pUC-yccM)
BW25113yccM::kan (pUC19)
BW25113 (pUC19)

(H)

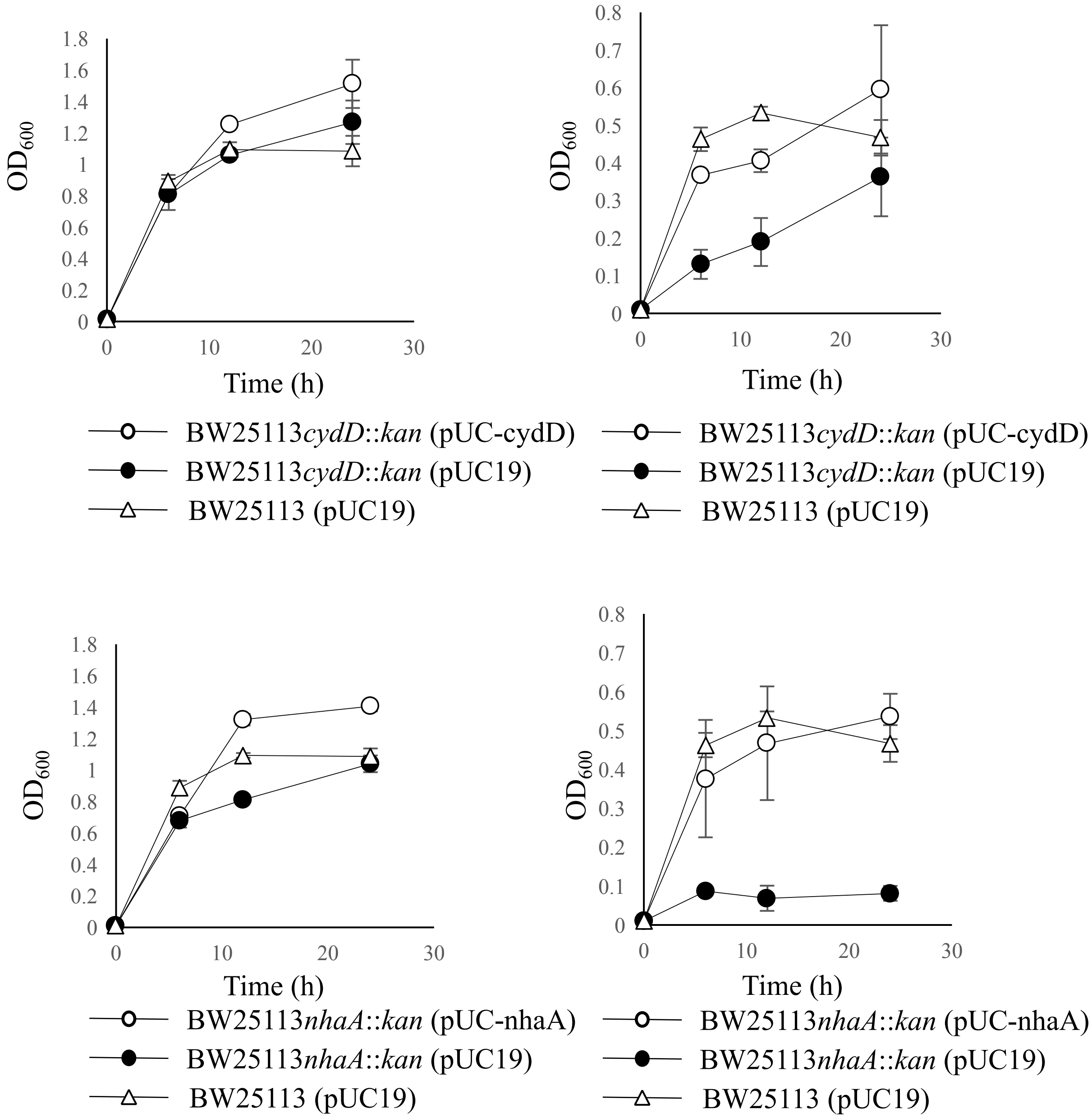

S5 Fig. Murata *et al*
