# Supplementary material for: Update of thermotolerant genes essential for survival at a critical high temperature in Escherichia coli
Source: PLoS One. 2018 Feb 27;13(2):e0189487. doi: 10.1371/journal.pone.0189487 (PMC5828445; doi:10.1371/journal.pone.0189487)
Supplement: S1 Table — (DOC) [file pone.0189487.s006.doc]

**S1 Table .** RT-PCR primers used in this study.

Name Sequence

atpC-5’ 5’-GGCAATGACTTACCACCTGGA-3’

atpC-3’ 5’-GTCAACTCGATAACGCGCAG-3’

atpD-5’ 5’-CTGGCCGGTACGGAAGTATC-3’

atpD-3’ 5’-CCCGGAGAACCGGTGAATAC-3’

atpG-5’ 5’-ACAAAGGCGTTCAATGCGAC-3’

atpG-3’ 5’-TGAGTAATGCTGGCCTGACG-3’

nuoE-5’ 5’-TGCACGAGAATCAACAACCAC-3’

nuoE-3’ 5’-TATACCGCTCCAGCAGTTCAG-3’

cydC-5’ 5’-GCCTCTGCCGTACGTATCTC-3’

cydC-3’ 5’-TCCGCCTTCACCTAACCAAC-3’

gntU-5' 5’-CCGCTGTTCTTTGAAGTGGC-3’

gntU-3' 5’-AGACCGTAAATCGCCACCAG-3’

phnO-5’ 5’-ATGCCTGCTTGTGAGCTTCG-3’

phnO-3’ 5’-GGAAGTGGCTCTGCTCGTAG-3’

yigP-5’ 5’-CCTTCCTGTATCGCTCACCC-3’

yigP-3’ 5’-CGTTTGGTCAGGGCATCAAC-3’

visC-5’ 5’-TGCCGCCAGCGAAAAATTAC-3’

visC-3’ 5’-AGCAAAGATGCGGATCGCTA-3’

argT-5’ 5’-CACCTACGCACCGTTCTCAT-3’

argT-3’ 5’-GAGCCAGCAAAGGCGAAATC-3’

yjgA-5’ 5’-TGACTAAGCAGCCCGAAGAC-3’

yjgA-3’ 5’-AAATCTGGCGTGCGGATTTC-3’

trpA-5’ 5’-TTCGTTCCTTTCGTCACGCT-3’

trpA-3’ 5’-ATGATTGAGGGGTAACGCGG-3’

yjiA-5’ 5’-CAGTTCGACCGTCTGGTCAT-3’

yjiA-3’ 5’-CGTCAATCCACAGCATCCCT-3’

yjiX-5' 5’-TGTTTGGTAACTTAGGACAGGC-3’

yjiX-3' 5’-ATACCGCCTTTACCATCGCC-3’

mdtG-5' 5’-CTATTACGCTCTGCCCTCGG-3’

mdtG-3' 5’-GTTACTGACGTTACCCGCCA-3’

nhaR-5’ 5’- TTACCGGACAGATTCGAGCG-3’

nhaR-3’ 5’- GCCCTAACATTGAACGTCGC-3’

fimH-5’ 5’- CATTCGCCTGTAAAACCGCC-3’

fimH-3’ 5’- CACGAGCAGAAACATCGCAG-3’

ydgI-5’ 5’- CACTCACCGCGCTGGTATTA-3’

ydgI-3’ 5’- CATCGCCAGCACAACAAACA-3’

yaiP-5’ 5’- CCTATGTTCCGCCCGATCAA-3’

yaiP-3’ 5’- TCCCAACGCCATAAAGCACA-3’

yraO-5’ 5’- TCAAACTCAAATTGCGGCGG-3’

yraO-3’ 5’- CACAGGCAATTTACCGTCAGC-3’
