# Supplementary material for: Update of thermotolerant genes essential for survival at a critical high temperature in Escherichia coli
Source: PLoS One. 2018 Feb 27;13(2):e0189487. doi: 10.1371/journal.pone.0189487 (PMC5828445; doi:10.1371/journal.pone.0189487)
Supplement: S2 Table — (DOC) [file pone.0189487.s007.doc]

**Supplemental Table 2.** Primers for gene cloning for complementation experiments.

Name Sequence

pUC19-5’ 5’-GATCCTCTAGAGTCGACCTG-3’

pUC19-3’ 5’- GATCCCCGGGTACCGAGCTC-3’

atpA-5’ 5’-cggtacccggggatcTTTATTCACCTGCGTGCCGT-3’

atpA-3’ 5’-cgactctagaggatcACCAGGATTGGGTTGCTTTGA-3’

gntK-5’ 5’-cggtacccggggatcGCCCACAAATTTGAAGTAGCTC-3’

gntK-3’ 5’- cgactctagaggatcGCATACGCGCCTTCATGACT-3’

lpxL-5’ 5’-cggtacccggggatcGTTCCTGCGAGATGGGAAAG-3’

lpxL-3’ 5’-cgactctagaggatcGCGCAATCCAGAGAGCTTTTA-3’

fimG-5’ 5’-cggtacccggggatcTTTTTACGCCCGGCTAATGG-3’

fimG-3’ 5’-cgactctagaggatcTTTCATTACAATCATCTCTTCGGGT-3’

yraN-5’ 5’-cggtacccggggatcCTACCGTTAATGCAGCAGGC-3’

yraN-3’ 5’-cgactctagaggatcAGCTTTAATTCTTTCTTGCACGCT-3’

yccM-5’ 5’-cggtacccggggatcATGAAATCGACATTGCCCGC-3’

yccM-3’ 5’-cgactctagaggatcTTGTGAACATCACCCCGTGC-3’

cydD-5’ 5’-cggtacccggggatcGGCGACACCATGCGACTAT-3’

cydD-3’ 5’-cgactctagaggatcCAGTGCCAGATAGGGTAGCA-3’

nhaA-5’ 5’-cggtacccggggatcCTAATCCTGAAGACGCCTCG-3’

nhaA-3’ 5’-cgactctagaggatcGAGCCGTTTATGGCTCCCCG-3’
